# Supplementary material for: Predicting visual function by interpreting a neuronal wiring diagram
Source: Nature. 2024 Oct 2;634(8032):113–23. doi: 10.1038/s41586-024-07953-5 (PMC11446822; doi:10.1038/s41586-024-07953-5)
Supplement: Supplementary file 6 — CRF and ERF predictions for individual TmY4 and TmY9 cells. Analogous to Supplementary Data 3, but for TmY target types. Shown are the top four monosynaptic pathways, the strongest pathway passing through each of the top ten intermediary types (ranking from Extended Data Fig. 7), and the trisynaptic pathway Tm1–TmY–Dm3–TmY (see the section entitled Prediction of spatial normalization). [file 41586_2024_7953_MOESM6_ESM.zip › DataS4/TmY9q/720575940632511274.pdf]

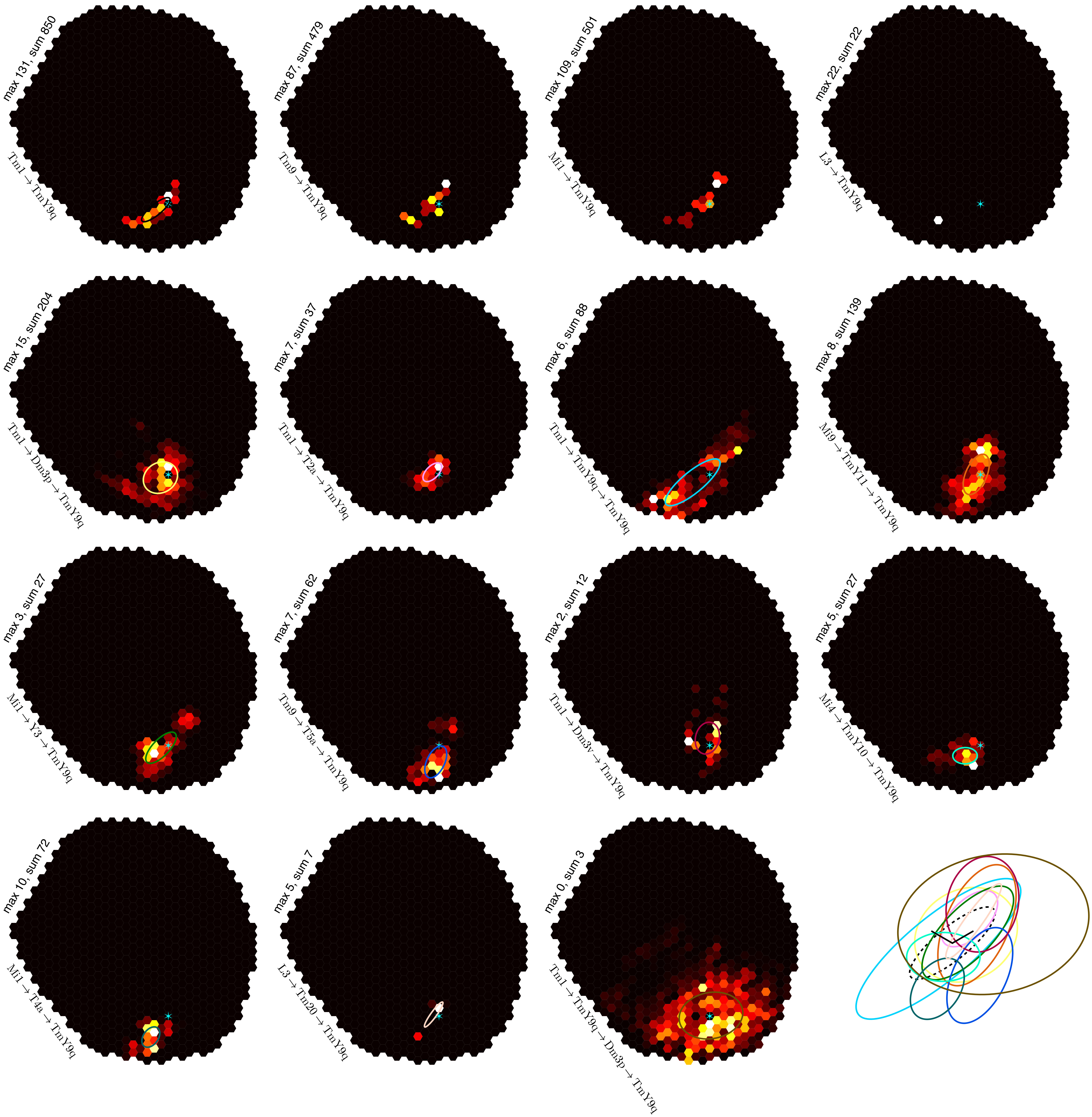
max 131, sum 850
Tm1 → TmY9q
max 87, sum 479
Tm9 → TmY9q
max 109, sum 501
Mi1 → TmY9q
max 22, sum 22
L3 → TmY9q
max 15, sum 204
Tm1 → Dm3p → TmY9q
max 7, sum 37
Tm1 → T2a → TmY9q
max 6, sum 88
Tm1 → TmY9q → TmY9q
max 8, sum 139
Mi9 → TmY11 → TmY9q
max 3, sum 27
Mi1 → Y3 → TmY9q
max 7, sum 62
Tm9 → T5a → TmY9q
max 2, sum 12
Tm1 → Dm3v → TmY9q
max 5, sum 27
Mi4 → TmY10 → TmY9q
max 10, sum 72
Mi1 → T4a → TmY9q
max 5, sum 7
L3 → Tm20 → TmY9q
max 0, sum 3
Tm1 → TmY9q → Dm3p → TmY9q
